# Vaccines for Autoimmune Diseases: Stopping Triggers, Restoring BalanceA Systematic Review with Qualitative Synthesis

**DOI:** 10.31138/mjr.111225.rba

**Published:** 2026-06-12

**Authors:** Jozélio Freire de Carvalho, Cezar Augusto Muniz Caldas, Yehuda Shoenfeld

**Affiliations:** 1 Institute for Health Sciences, Federal University of Bahia, Salvador, Bahia, Brazil;; 2 Faculty of Medicine, Federal University of Pará, Belém Pará, Brazil;; 3 Reichmann University, Herzelia, Israel and Zabludowicz Center for Autoimmune Diseases, Sheba Medical Center, Tel Hashomer, Israel

**Keywords:** autoimmune diseases, vaccines, tolerogenic vaccines, Epstein-Barr virus, immune tolerance, streptococcus pyogenes

## Abstract

**Objective:**

To critically review the scientific background on vaccines designed to prevent autoimmune diseases, including two main strategies: (1) vaccines directed against pathogens involved in triggering disease, including Epstein-Barr virus (EBV), enterovirus and Streptococcus pyogenes; and (2) tolerogenic vaccine approaches targeting the immune response to restore tolerance to self-antigens.

**Methods:**

We performed a thorough systematic review of clinical trials, observational studies, meta-analysis and pre-clinical data. A broad search was implemented in biomedical databases and gray literature. This systematic review was conducted with qualitative synthesis, given the heterogeneity of study designs, diseases, and outcomes. Two reviewers independently selected studies for inclusion and extracted data, using standard risk of bias and quality of evidence assessment tools.

**Results:**

There were 68 studies, 36 in humans and 32 preclinical studies included. In the anti-infective area, options include gp350 vaccine for EBV (phase 2), mRNA based early-stage vaccines, a population-based study of the impact of rotavirus vaccination on type 1 diabetes incidence, and inactivated PRV-101 Coxsackie B strain vaccine; for rheumatic fever, phase 1 trials are providing evidence with vaccines such as StreptAnova and J8/S2. With regard to the tolerogenic line, tolerogenic DC-based and of myelin peptides vaccines have shown immune regulation in illnesses such as MS and RA, but do not yet demonstrate solid clinical evidence.

**Conclusions:**

Trigger infections vaccines are now nearing clinical use whereas tolerogenic vaccines constitute an attractive approach for the future. The combination of these approaches has the potential to inform future preventive and therapeutic strategies, but current evidence remains heterogeneous and largely early-stage, particularly for tolerogenic platforms.

## INTRODUCTION

Autoimmune diseases affect 5–7% of the global population and represent a major public health problem with respect to disability, healthcare utilisation, and costs for healthcare systems.^1^ Type 1 diabetes (T1D), rheumatoid arthritis (RA), systemic lupus erythematosus (SLE), and multiple sclerosis (MS) are four leading autoimmune diseases in humans. When the immune system loses its ability to preserve natural tolerance, it mistakenly attacks the body’s own tissues and becomes chronically inflamed, resulting in progressive damage.

In recent decades, progress in the treatment of autoimmune diseases has been achieved through conventional immunosuppressive drugs and biologic therapies. These treatments have increased survival rates and improved quality of life in many cases. Nevertheless, they fail to target the origins of autoimmunity and are commonly accompanied by serious side effects, including greater susceptibility to infections, increased risk of cancer, and toxicity.^2^

The development of autoimmune diseases involves a complex interplay between genetic susceptibility and environmental triggers.^3^ Infection is also considered one of the key environmental factors in initiating auto-immunity by breaking immune tolerance. This can be explained by three principal mechanisms: molecular mimicry, bystander activation, and epitope spreading.^4^

A pivotal prospective study involving approximately 10 million U.S. armed forces members demonstrated the onset of multiple sclerosis (MS) after Epstein–Barr virus (EBV) infection, with a 32-fold higher risk of progression following EBV seroconversion.^5^ These results clearly demonstrate the potential value of blocking specific infections as an upstream strategy to control autoimmune disease.

Two fundamental vaccination approaches to combat this problem have been described by investigators. The first strategy focuses on preventing infections that may trigger autoimmune disease. Currently, studies are underway to develop vaccines against EBV for the prevention of MS, enteroviruses for protection against T1D, and Streptococcus pyogenes for rheumatic fever.^5^ The second approach involves tolerogenic vaccines aimed at redirecting the immune system following controlled exposure to specific autoantigens. This strategy induces tolerogenic immune responses, re-establishing lost tolerance without compromising the organism’s immunoprotection against pathogens and tumours.^6,7^ In this review, we use the term “vaccine” broadly to include both prophylactic pathogen-targeting vaccines and antigen-specific tolerogenic immunisation strategies intended to induce immune tolerance, while distinguishing these approaches mechanistically and by their translational stage.

This review summarises the current evidence on both infection-preventing and tolerance-restoring vaccines, while highlighting existing knowledge gaps and identifying potential directions for future research.

**Figure 1.**
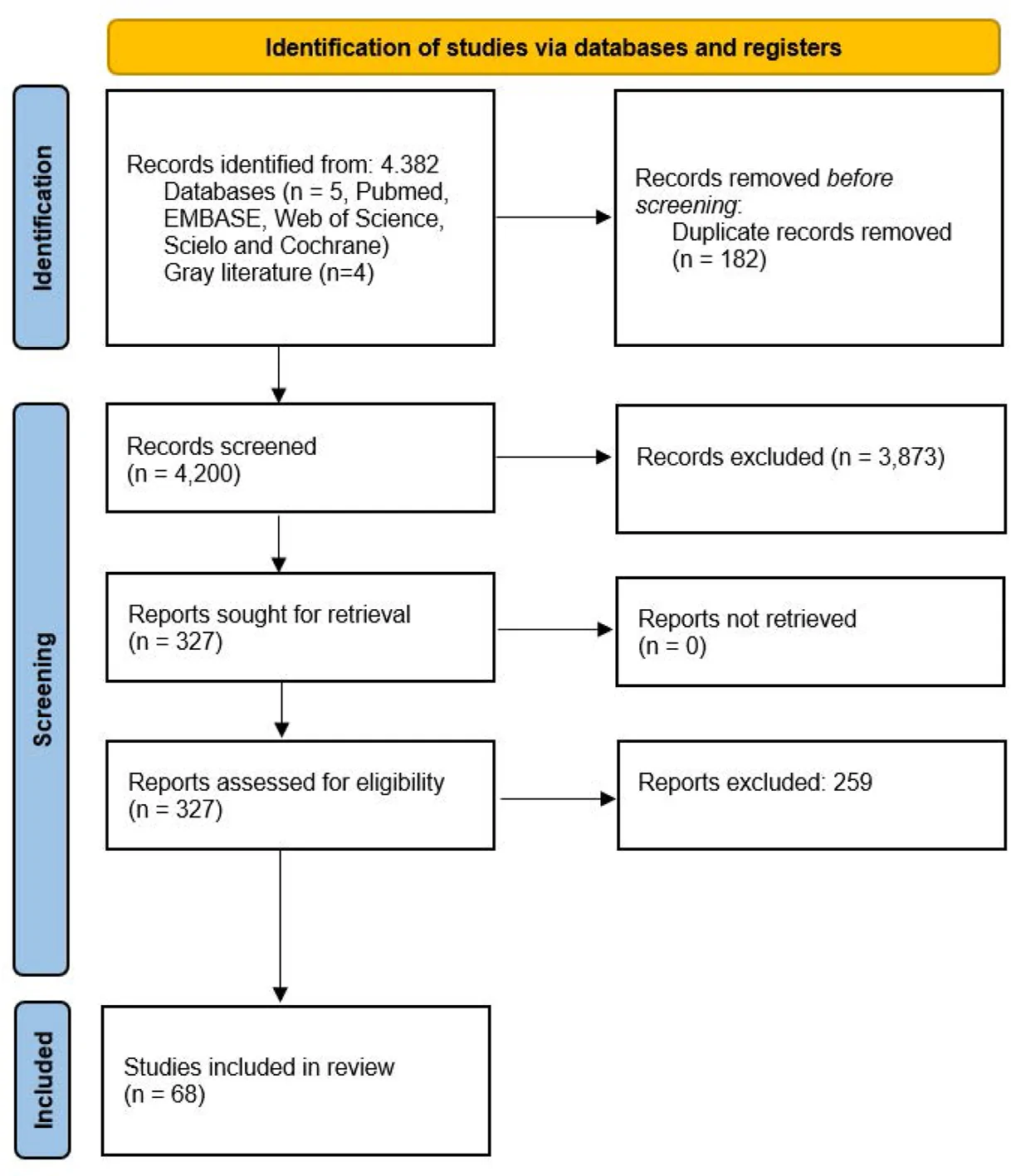
Flowchart of the included studies.

## METHODS

### Search strategy

A comprehensive search was conducted in the PubMed/MEDLINE, Embase, Cochrane Library, Web of Science, SciELO, and gray literature sources (Clinical-Trials.gov, medRxiv, CNKI, Wanfang). The search period covered January 2000 to September 2025, with no language restrictions. Combined terms such as autoimmune disease, vaccine, tolerogenic, Epstein-Barr virus, enterovirus, rotavirus, group A Streptococcus, multiple sclerosis, type 1 diabetes, rheumatoid arthritis, among others, were used. The review was conducted and reported according to the PRISMA 2020 statement.^8^ A PRISMA flow diagram was included to summarise the study selection process, including reasons for full-text exclusions.

### Eligibility Criteria

The research included clinical trials, observational studies, case series, systematic reviews and meta-analyses of vaccines that prevent or modify autoimmune diseases. Exclusively preclinical studies were included only for mechanistic contextualisation. The research team excluded studies which contained insufficient data or duplicate information or inadequate methodological descriptions. At the full-text stage, studies were excluded mainly due to: (i) absence of vaccine-related intervention (e.g., purely immunosuppressive biologics or non–vaccine-based therapies), (ii) outcomes not related to autoimmune diseases or immune tolerance, (iii) insufficient methodological description or incomplete reporting of results, and (iv) duplicate publications or overlapping cohorts.

### Study Selection and Data Extraction

Two independent reviewers performed the title and abstract screening before conducting full-text reviews to establish study eligibility. The reviewers solved any disagreements by reaching consensus or by asking a third reviewer for input. The researchers extracted information about six key variables which included study population, vaccine type, study design, clinical and immunological results, adverse effects, methodological quality and development stage. Due to the heterogeneity of included diseases, study designs, and endpoints, a quantitative meta-analysis was not feasible; therefore, results were synthesised qualitatively.

### Methodological Quality Assessment

The risk of bias was assessed using RoB 2 for randomised clinical trials^9^ and the Newcastle-Ottawa Scale (NOS) for observational studies.^10^ The certainty of evidence was graded using the GRADE system.^11^

## RESULTS

The systematic search identified 4,382 records from databases and gray literature sources. The assessment of 327 articles proceeded through duplicate elimination and title and abstract evaluation. Of these, 68 studies were included in the final synthesis. The research included 36 human studies, which made up 53% of the total number of studies. Among them, 36 (53%) were conducted in humans, including 18 phase 1 or 2 clinical trials, 11 population-based observational studies, and 7 systematic reviews or meta-analyses. The articles were split into two primary sections which included vaccines for infectious triggers and tolerogenic vaccines for people with established autoimmunity or those at high risk.^12^

The phase 2 clinical trial of the recombinant gp350 vaccine evaluated its safety in 181 EBV-seronegative young adults but demonstrated 78% effectiveness in stopping infectious mononucleosis although it failed to stop subclinical infections. The treatment proved to be safe because patients only developed mild adverse effects which disappeared spontaneously.^13^ The most advanced vaccine candidates, including mRNA-based vaccines **(**mRNA-1189 and mRNA-1195), are in phase 1 trials, but researchers have only reported safety and immunogenicity results without any clinical efficacy data**.**^14^ There is still no direct evidence that preventing EBV infection reduces the incidence of MS, and large, long-term multicentre trials are needed to confirm this hypothesis.

Regarding type 1 diabetes (T1D), population-based studies have explored the association between rotavirus vaccination and T1D incidence, suggesting a potential reduction in disease occurrence following national immunisation programs.^15,16^ However, heterogeneity across studies (including differences in vaccination coverage, genetic background, and environmental exposures) limits causal inference and supports cautious interpretation.

For enteroviruses, particularly Coxsackie B, mechanistic and translational evidence has supported their association with T1D, including viral detection in pancreatic tissue.^17^ The inactivated PRV-101 Coxsackie B strain vaccine has advanced into early-phase clinical development, with available safety and immunogenicity data but no confirmed impact on autoimmune disease incidence.^18^

For rheumatic fever, an autoimmune disease triggered by Streptococcus pyogenes infection, phase 1 trials have provided preliminary evidence supporting vaccine candidates such as StreptAnova and multivalent peptide-based formulations including J8/S2 and P*17/S2.^19,20^ The World Health Organisation has emphasised the need for rigorous safety monitoring throughout development, considering historical safety concerns related to older S. pyogenes vaccine approaches.^21^

In MS, antigen-specific tolerogenic vaccination approaches have focused on myelin-related peptides and immune tolerisation strategies. Clinical studies have tested peptide-based vaccines such as ATX-MS-1467, showing immune modulation and acceptable safety profiles, although durable clinical benefit remains uncertain**.**^22,23^ Preclinical studies evaluating tolerogenic platforms and alternative delivery systems were also identified, supporting mechanistic plausibility for restoring immune tolerance.^24^

For T1D, the GAD65 enzyme-based vaccine was tested in over 500 newly diagnosed children and adolescents. Although it induced specific immune responses, there was no significant preservation of pancreatic beta-cell function in phase 3 trials.^25^ The DiaPep277 peptide, derived from HSP60, showed encouraging early results but failed to confirm efficacy in subsequent studies, leading to the discontinuation of its development.^26^ Currently, research in animal models has explored immunomodulatory nanoparticles, with encouraging preliminary results, although still limited to the preclinical phase.^27^

In rheumatoid arthritis, vaccines using tolerogenic dendritic cells (tolDC) were tested in phase 1 trials. The AuToDeCRA study, which evaluated intra-articular administration of these cells, demonstrated safety, good patient acceptance, and increased levels of regulatory cytokines, such as IL-10.^28^ Another strategy, based on citrullinated peptides, showed potential to modulate autoreactive T cells in individuals carrying high-risk HLA alleles, providing a scientific rationale for future clinical studies.^29^

In SLE, phase 1 studies investigated vaccines composed of irradiated autologous T cells. The results showed a transient reduction in anti-DNA autoantibody titres, with no severe adverse events, although the effects were temporary.^30,31^ For myasthenia gravis, no human clinical trials were identified, with evidence remaining limited to preclinical models.

See **Table 2** for a summary of the included studies on tolerogenic vaccines.

**Table 1.** Vaccines of infectious agents for autoimmune diseases.

**Target Disease**	**Vaccine Type**	**Development Stage**	**Main Clinical Results**	**References**
Multiple Sclerosis (MS)	Recombinant gp350 (EBV)	Phase 2	78% reduction in infectious mononucleosis, no prevention of subclinical infection	[4,11]
Multiple Sclerosis (MS)	mRNA-1189 and mRNA-1195 (EBV)	Phase 1	Safety and strong immunogenicity demonstrated, no clinical efficacy data yet	[12]
Type 1 Diabetes (T1D)	Rotavirus vaccine	Population-level implementation	Modest reduction in T1D incidence in countries with high vaccination coverage	[13,14]
Type 1 Diabetes (T1D)	PRV-101 (Coxsackie B)	Phase 1 completed, Phase 2/3 planned	Demonstrated safety, neutralizing antibodies in >90% of vaccinated participants	[15,16]
Rheumatic Fever	StreptAnova (30-valent M protein)	Phase 1	Robust immune response, no serious adverse events	[17]
Rheumatic Fever	Peptide vaccines (J8/S2 and P*17/S2)	Early phase	Promising immunogenicity, focus on preventing molecular mimicry	[18,19]
Guillain-Barré Syndrome	*Campylobacter jejuni* (mimetic epitopes)	Pre-clinical	Data restricted to animal models and in vitro studies	[34,35]

**Table 2.** Tolerogenic vaccines for autoimmune diseases.

**Autoimmune Disease**	**Mechanism**	**Stage**	**Main Findings**	**References**
Multiple Sclerosis (MS)	Myelin peptides (ATX-MS-1467)	Phase 1/2	Transient reduction of inflammatory lesions on MRI, acceptable safety profile	[36,37 ]
Multiple Sclerosis (MS)	Autologous cells coupled with myelin peptides	Pre-clinical / Early phase	Selective modulation of autoreactive T cells	[5]
Type 1 Diabetes (T1D)	GAD65-based vaccine	Phase 3	Induced specific immune response without preserving beta-cell function	[40]
Type 1 Diabetes (T1D)	DiaPep277 (HSP60)	Phase 2/3	Initial positive results, failed to confirm efficacy in advanced trials	[41]
Type 1 Diabetes (T1D)	Immunomodulatory nanoparticles	Pre-clinical	Promising results in animal models, not yet tested in humans	[42]
Rheumatoid Arthritis (RA)	Tolerogenic dendritic cells (tolDC)	Phase 1	Adequate safety and increased regulatory cytokines	[43]
Rheumatoid Arthritis (RA)	Citrullinated peptides	Pre-clinical	Potential to modulate autoreactive T cells in high-risk HLA individuals	[6]
Systemic Lupus Erythematosus (SLE)	Irradiated autologous T cells	Phase 1	Transient reduction of anti-DNA autoantibody titers	[45]
Myasthenia Gravis	Experimental vaccines in animal models	Pre-clinical	Early promising evidence, not yet tested in humans	[46]

When classifying the studies by stage of development, it was observed that the most innovative vaccines are still predominantly in the preclinical phase. This category includes immunomodulatory nanoparticles for T1D and SLE, vaccines against Campylobacter jejuni, and early cellular approaches.^32–34^ In the early phase in healthy humans are vaccines such as PRV-101, mRNA-based vaccines against EBV, StreptAnova, and peptide vaccines J8/S2 and P*17/S2. In clinical trials involving patients with autoimmune diseases are the ATX-MS-1467 vaccine for MS, GAD65 for T1D, tolDC for rheumatoid arthritis, and irradiated autologous T cells for SLE. This stratification highlights that although there are promising proposals, few have reached the stage of evaluation in target populations, while most remain at experimental stages (see **Table 3**).

**Table 3.** Development pipeline and evidence strength of the vaccines.

**Vaccine Type / Target**	**Development Stage**	**Quality of Evidence (GRADE)**	**Main Diseases Targeted**
Recombinant gp350 (EBV)	Phase 2	Moderate	Multiple Sclerosis
mRNA-1189 / mRNA-1195 (EBV)	Phase 1	Low	Multiple Sclerosis
Rotavirus vaccine	Implemented at population level	Moderate	Type 1 Diabetes
PRV-101 (Coxsackie B)	Phase 1 completed	Low	Type 1 Diabetes
StreptAnova (M protein)	Phase 1	Low	Rheumatic Fever
J8/S2 and P*17/S2 peptides	Early phase	Low	Rheumatic Fever
*Campylobacter jejuni* epitope vaccine	Pre-clinical	Very Low	Guillain-Barré Syndrome
ATX-MS-1467 (myelin peptides)	Phase 1/2	Very Low	Multiple Sclerosis
Autologous cells + myelin peptides	Pre-clinical / Early phase	Very Low	Multiple Sclerosis
GAD65-based vaccine	Phase 3	Low	Type 1 Diabetes
DiaPep277 (HSP60)	Phase 2/3	Low	Type 1 Diabetes
Immunomodulatory nanoparticles	Pre-clinical	Very Low	Type 1 Diabetes, Lupus
tolDC (tolerogenic dendritic cells)	Phase 1	Very Low	Rheumatoid Arthritis
Citrullinated peptides	Pre-clinical	Very Low	Rheumatoid Arthritis
Irradiated autologous T cells	Phase 1	Very Low	Systemic Lupus Erythematosus
Experimental vaccines for MG	Pre-clinical	Very Low	Myasthenia Gravis

The methodological quality assessment revealed wide variability among the included studies. Using the RoB 2 tool for clinical trials, eight studies (44%) were rated as having low risk of bias, seven (39%) moderate risk, mainly due to lack of complete blinding, and three (17%) high risk, due to loss to follow-up and selective outcome reporting. Among observational studies assessed using the Newcastle-Ottawa Scale, six (55%) were classified as high quality (≥7 points), three (27%) as intermediate quality (5–6 points), and two (18%) as low quality (<5 points). The GRADE methodology rated the certainty of evidence as moderate for anti-EBV vaccines and rotavirus vaccination in T1D, low for S. pyogenes and Coxsackie B vaccines, and very low for tolerogenic vaccines, reflecting their early development stage and small sample sizes. These findings reinforce the need for multicentre, randomised trials with robust clinical outcomes to consolidate the role of vaccines in preventing autoimmune diseases.

## DISCUSSION

This systematic review focuses on two complementary vaccine strategies for preventing the onset of auto-immune diseases and potentially modulating disease course. The first strategy relies on vaccines against pathogens to prevent loss of immune tolerance through mechanisms such as molecular mimicry, non-specific immune activation, and epitope spreading.^4^ The second approach—clinical tolerance induction through tolerogenic vaccines—aims to re-establish tolerance by controlled autoantigen presentation in immunomodulatory settings, thereby reducing autoimmune tissue damage.^6,7^ Rather than reiterating descriptive characteristics of individual vaccine platforms, the discussion below integrates mechanistic plausibility, translational maturity, and clinical implications of these approaches. Given the breadth of diseases, endpoints, and study designs included, our conclusions should be interpreted as qualitative and hypothesis-generating, rather than disease-specific estimates of effect.

Research on vaccines against infection triggers confirms that specific pathogens play a central role in initiating autoimmune responses. The association between *Streptococcus pyogenes* and rheumatic fever is a paradigmatic example, in which molecular mimicry between streptococcal antigens and cardiac tissue leads to cross-reactive immune responses.^19,20^ In this context, vaccine development should be viewed not merely as infection prevention, but as interruption of a well-characterised autoimmune cascade**.** Of infectious agents, EBV exhibits the strongest association with MS, with prospective cohort studies demonstrating that virtually all patients who develop MS have prior EBV infection, and that risk markedly increases following primary infection.^5^

Beyond epidemiological associations, recent mechanistic studies have provided a direct immunopathological link between EBV infection and CNS autoimmunity**.** Lanz et al. demonstrated that clonally expanded B cells in MS recognise both EBV nuclear antigen 1 (EBNA1) and the glial protein GlialCAM, and that immunisation with EBNA1-derived epitopes can induce autoimmune demyelination in experimental models.^35^ These findings strengthen the biological rationale for EBV-targeted vaccination as a preventive strategy, while not constituting proof of disease prevention in humans. Accordingly, although gp350-based and mRNA-based EBV vaccines have demonstrated immunogenicity and acceptable safety profiles.^13,14^ their capacity to reduce MS incidence remains hypothetical and requires long-term, population-based efficacy trials.

Recent high-impact translational research has further expanded the conceptual framework linking viral exposure to long-term immune dysregulation. In this context, Younis et al. introduced the concept of immune imprinting, demonstrating that early viral encounters can durably shape adaptive immune responses and influence susceptibility to immune-mediated diseases.^36^ Although not designed as a vaccine or prevention trial, this work provides strong biological support for the rationale that upstream immunological interventions—such as pathogen-targeted vaccination—may modify autoimmune risk trajectories when implemented before the establishment of autoreactive immune memory and epitope spreading.

Current data also suggest that rotavirus vaccination may confer a modest protective effect against type 1 diabetes (T1D). Population-based studies and meta-analyses consistently report an inverse association between rotavirus immunisation and T1D incidence, although effect sizes vary according to genetic background, environmental exposure, and vaccination coverage.^15,16^ Importantly, these observations should be interpreted as associative rather than causative, and do not exclude the contribution of other enteroviruses in disease initiation. Indeed, enteroviral persistence— particularly Coxsackie B—has been documented in pancreatic tissue of patients with recent-onset T1D,^17^ supporting ongoing interest in enterovirus-targeted vaccines. The PRV-101 vaccine represents a translational milestone in this field; however, its potential to prevent autoimmune diabetes remains to be demonstrated in adequately powered clinical trials.^18^

**Figure 2.**
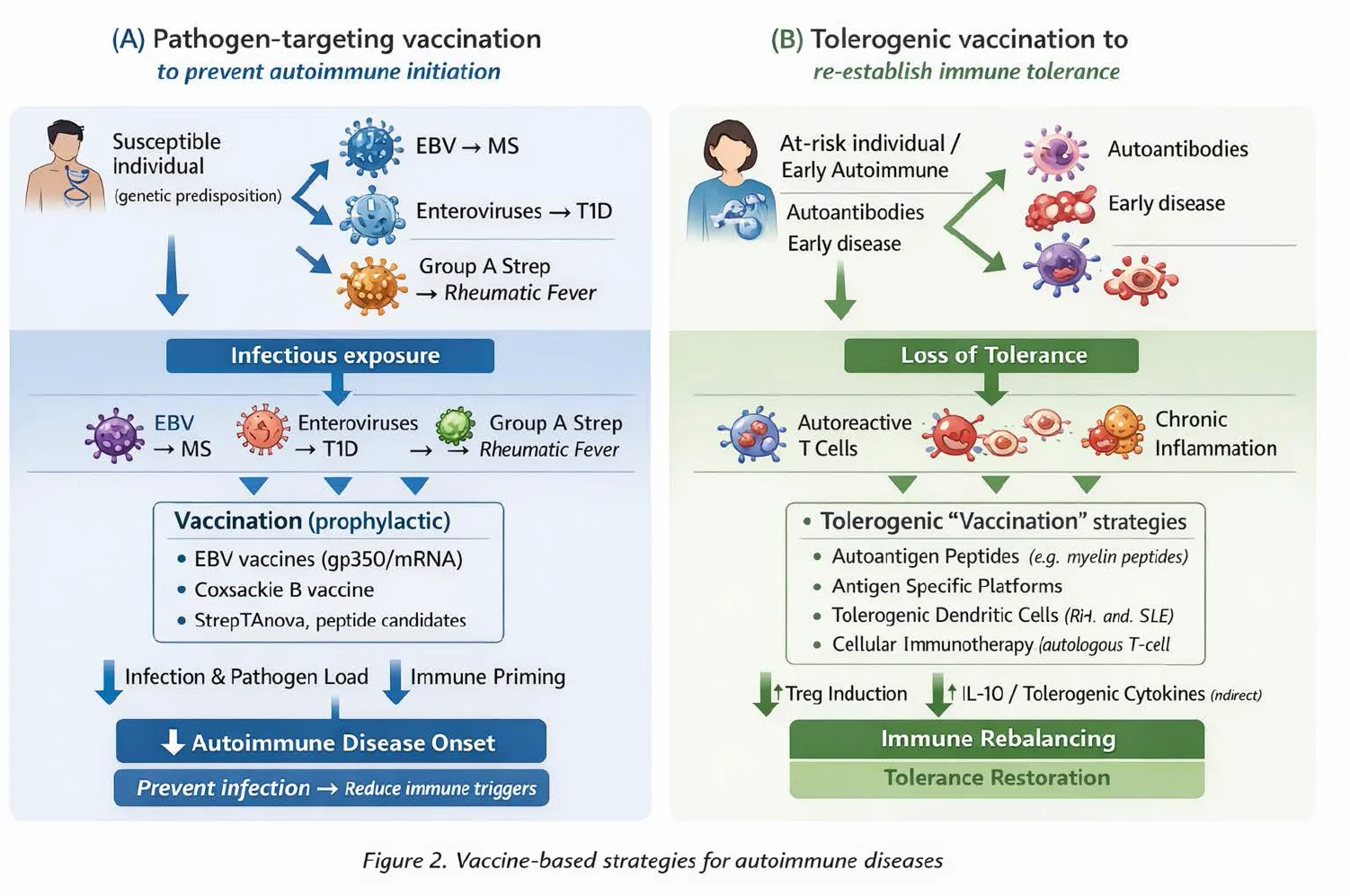
Mechanistic overview of vaccine-based strategies for autoimmune diseases: **(A)** pathogen-targeting vaccination and **(B)** tolerogenic vaccination. **(A)** Pathogen-targeting vaccination aims to prevent infection-related immune triggers implicated in autoimmune disease initiation (e.g., Epstein–Barr virus [EBV], enteroviruses, and Group A *Streptococcus*), thereby reducing downstream mechanisms such as molecular mimicry, bystander activation, and epitope spreading. **(B)** Tolerogenic vaccination aims to restore immune tolerance through controlled presentation of autoantigens in immunomodulatory contexts (e.g., autoantigen peptides, antigen-specific platforms, tolerogenic dendritic cells, and selected cellular immunotherapy approaches), promoting regulatory immune responses while avoiding broad immunosuppression. Together, these strategies represent complementary translational pathways for autoimmune disease prevention and immune rebalancing. EBV, Epstein–Barr virus; GAS, Group A *Streptococcus*; IL-10, interleukin-10; MS, multiple sclerosis; RA, rheumatoid arthritis; SLE, systemic lupus erythematosus; T1D, type 1 diabetes; Treg, regulatory T cell.

Vaccines against *S. pyogenes* also hold promise, particularly in high-burden regions where rheumatic fever and rheumatic heart disease remain major public health problems. Early-phase studies of candidates such as StreptAnova and peptide-based vaccines (J8/S2 and P*17/S2) have demonstrated immunogenicity and acceptable short-term safety.^19,20^ Given historical concerns regarding vaccine-induced autoimmunity in this context, stringent safety surveillance remains essential throughout clinical development, as emphasised by the World Health Organisation.^21^

Adjuvant selection represents another critical aspect in vaccine development for autoimmune-prone populations. Classical aluminium-based adjuvants are increasingly being optimised or replaced, in part due to concerns regarding excessive innate immune activation in genetically susceptible individuals. Contemporary adjuvant systems, including AS04, MF59, CpG oligonucleotides, virosomes, and lipid nanoparticles, aim to balance immunogenicity with immune regulation, minimising unnecessary inflammatory signals.^37,38^

These refinements are particularly relevant when vaccines are intended for large-scale preventive use. Tolerogenic vaccines present a fundamentally different and more complex challenge. Unlike pathogen-targeting vaccines, they seek to modulate an already dysregulated immune system. In MS, myelin-derived peptide vaccines such as ATX-MS-1467 have demonstrated antigen-specific immune modulation and transient reductions in inflammatory activity, without major safety concerns.^22,23^ However, durable clinical benefit has not yet been established, underscoring the distinction between immunological proof-of-concept and long-term disease modification. Similar limitations apply to tolerogenic strategies in T1D, where GAD65- and DiaPep277-based vaccines induced immune responses but failed to preserve β-cell function in phase 3 trials.^25,26^

In rheumatoid arthritis, tolerogenic dendritic cell– based approaches have shown acceptable safety and induction of regulatory cytokine profiles in early-phase trials, suggesting potential utility in selected patient subsets or early disease stages.^28^ Likewise, citrullinated peptide–based strategies offer a biologically compelling approach in genetically predisposed individuals, although clinical validation remains preliminary.^29^ Across diseases, tolerogenic vaccines appear most promising when applied early in the autoimmune process, before extensive epitope spreading and irreversible tissue damage occur.^39,40^

Developmental staging analysis reveals a pronounced translational gap. Most tolerogenic platforms remain confined to preclinical or early-phase clinical studies, whereas infection-targeting vaccines have advanced further along the clinical pipeline. This discrepancy reflects not only technical complexity, but also regulatory, ethical, and biomarker-related challenges inherent to immune tolerance induction. Continued investment in translational research and validated immunological endpoints will be essential to bridge this gap.

Finally, several limitations warrant consideration. The heterogeneity of diseases, vaccine platforms, and outcomes precluded quantitative meta-analysis. Many early-phase trials are subject to moderate or high risk of bias, and preclinical literature may overrepresent positive findings. Accordingly, the present review should be interpreted as an integrative synthesis rather than a definitive assessment of efficacy. Its principal contribution lies in framing infection-preventive and tolerance-restoring vaccination strategies within a unified translational perspective.

## CONCLUSION

This systematic review summarised two vaccine approaches for the prevention and control of autoimmune diseases by preventing infections triggers and restoring immune tolerance. The infection-trigger approach targets pathogens which play a role in the initiation of autoimmunity, including EBV in MS, enteroviruses in T1D, and S. pyogenes in rheumatic fever. While no direct evidence exists that vaccinations against EBV and enteroviruses will prevent autoimmune diseases, the advancement of the gp350 vaccine and mRNA-based EBV vaccines, PRV-101 vaccine for Coxsackie B, and S. pyogenes vaccine candidates (StreptAnova and J8/S2, P*17/S2) represent significant milestones in early-stage translational research.

Tolerogenic vaccines, including those based on autoantigens, peptide immunisation, tolerogenic dendritic cells, and cellular approaches, represent an emerging strategy aimed at restoring immune tolerance in genetically predisposed individuals or patients with established disease. However, current clinical evidence remains limited, with most studies confined to early-phase trials or preclinical settings, and with outcomes largely centred on immunologic modulation rather than durable clinical endpoints.

Given the heterogeneity of diseases, platforms, and endpoints across the included studies, our findings should be interpreted as a qualitative synthesis rather than disease-specific estimates of efficacy. Accordingly, the novelty of this work lies in its integrative and translational perspective—bringing together trigger-preventing and tolerance-restoring strategies within a unified framework—rather than in introducing a fundamentally new prevention paradigm.

Although significant challenges remain—including methodological limitations, risk of bias in early-phase trials, potential publication bias in preclinical research, and the need for long-term efficacy studies—vaccines targeting infectious triggers and tolerogenic vaccination strategies together may contribute to future preventive and therapeutic advances in autoimmunity. Continued multidisciplinary collaboration and robust clinical trials will be essential to define the role of these approaches in reducing the global burden of autoimmune diseases.

## Data Availability

All data are available upon request.
